# Patient experiences using public and private insurance coverage for abortion in Illinois: Implementation successes and remaining gaps

**DOI:** 10.1111/psrh.12259

**Published:** 2024-04-11

**Authors:** Madeline Quasebarth, Madeleine Boesche, Tecora Turner, Amy Moore, Danielle Young, Debra Stulberg, Lee Hasselbacher

**Affiliations:** ^1^ Ci3, Department of Obstetrics and Gynecology University of Chicago Chicago Illinois USA; ^2^ Crown Family School of Social Work, Policy, and Practice University of Chicago Chicago Illinois USA; ^3^ Pritzker School of Medicine University of Chicago Chicago Illinois USA; ^4^ Planned Parenthood of Illinois Chicago Illinois USA; ^5^ Department of Family Medicine University of Chicago Chicago Illinois USA

**Keywords:** abortion, domestic, law/legal issues, policy, qualitative research methods, United States

## Abstract

**Context:**

Insurance coverage for abortion in states where care remains legal can alleviate financial burdens for patients and increase access. Recent policy changes in Illinois required Medicaid and some private insurance plans to cover abortion care. This study explores policy implementation from the perspectives of patients using their insurance to obtain early abortion care.

**Methodology:**

Between July 2021 and February 2022, we interviewed Illinois residents who recently sought abortion care at ≤11 weeks of pregnancy. We also interviewed nine key informants with experience providing or billing for abortion or supporting insurance policy implementation in Illinois. We coded interview transcripts in Dedoose and developed code summaries to identify salient themes across interviews.

**Results:**

Most participants insured by Illinois Medicaid or eligible for enrollment received full coverage for their abortions; most with private insurance did not and faced challenges learning about coverage status. Some opted not to use insurance, often citing privacy concerns. Participants who benefited from abortion coverage expressed relief, gave examples of other financial challenges they could prioritize, and described feeling in control of their abortion experience. Those without coverage described feeling stressed, uncertain, and constrained in their decision‐making.

**Conclusion:**

When abortion was fully covered by insurance, it reduced financial burdens and enhanced reproductive autonomy. Illinois Medicaid policy—with seamless enrollment options and appropriate reimbursement rates—offers a model for improving abortion access in other states. Further investigation is needed to determine compliance among private insurance companies and increase transparency.

## INTRODUCTION

With the overturn of *Roe v. Wade* and the proliferation of restrictions on abortion across the country, Illinois has emerged as a destination for access to abortion care in the United States, particularly in the Midwest. Illinois providers and abortion funds are now inundated with people seeking abortion care from across the country; recent surveillance data indicates that Illinois is the highest surge state in the 12 months post‐*Dobbs*.^1^ With resources stretched to support access, it is critical to understand the efficacy of Illinois state policies aimed at ameliorating financial barriers to abortion care.^1^, ^2^, ^3^ For example, the successful implementation of insurance‐related policies intended to reduce out‐of‐pocket costs for some patients can free up resources to support those without insurance coverage or those traveling to Illinois for care.^4^

In 2018, Illinois passed HB40, a policy requiring Medicaid coverage for abortion care.^5^ The next year, the state passed another law requiring private insurance plans to cover abortion if they covered other pregnancy‐related health care.^5^, ^6^ Illinois is one of five states and the only non‐coastal state to require private insurance plans to cover abortion similar to other pregnancy‐related care; it is also one of 17 states that cover abortion through Medicaid and only one of two states in the Midwest to do so.^6^, ^7^ In many states, Medicaid only covers abortion care under exceptions outlined in the federal Hyde Amendment: situations involving rape, incest, or threats to the life of the pregnant person.^8^ The Hyde Amendment restrictions create financial obstacles for low‐income people seeking abortion, and especially affect Black and Brown birthing people as they are more likely to be enrolled in Medicaid due to systemic racism and historical economic injustices.^4^, ^9^, ^10^, ^11^, ^12^, ^13^ In Illinois, most Medicaid enrollees must choose a plan offered through a managed care program; however, as of November 2019, all abortion claims must be billed directly to the state in a fee‐for‐service model (previously, managed care programs reimbursed for Hyde‐exception abortion care).^14^

Previous research in Illinois on the implementation of Medicaid coverage for abortion revealed significant challenges early on, many of which have been addressed through advocacy and state agency action.^7^ Illinois now offers appropriate reimbursement rates without unreasonable delay, allows providers to enroll patients in Medicaid the day of their appointment using the presumptive eligibility process permitted for pregnant people, and has a system to submit and track electronic claims.^7^, ^15^ Providers and other community stakeholders in Illinois have also reported that Medicaid coverage seemed to remove significant financial barriers for patients and allow for financial assistance resources to be reallocated to those without insurance coverage.^4^ For instance, one recent study at a single academic medical center found reduced patient costs following HB40.^15^ At the same time, stakeholders described some populations who may not be benefiting from Medicaid coverage, including minors, undocumented individuals, and those in rural areas.^4^, ^7^, ^16^ There is no research addressing the implementation of private insurance coverage requirements in Illinois, but research from other contexts suggests that this coverage can be confusing and incomplete in states where coverage is not entirely prohibited.^16^, ^17^, ^18^

Successfully expanded insurance coverage can reduce the cost of abortion care for qualifying individuals living in Illinois, which can improve equitable access.^7^, ^11^, ^16^, ^19^ In a survey of 5930 abortion patients 82% of participants described difficulties paying for abortion care.^20^ In 2022, for a single‐person household, this annual income equated to roughly $14,580. An abortion prior to 13‐weeks can cost between $475 and $575, upwards of 41% of an individual's monthly earnings, and may prove to be a catastrophic health expense for some.^21^ Past research has pointed to expanding health insurance coverage as a way to reduce delays in abortion care for those living in poverty; insurance coverage can also preserve household funds for other essential items (e.g., housing, food, etc.).^10^, ^11^, ^15^, ^17^, ^18^, ^19^ Expanding coverage and making abortion more affordable could also lead to an increase in reproductive autonomy, defined as an individual's complete empowerment to access full‐spectrum reproductive healthcare free from coercion or obstruction.^22^, ^23^, ^24^

Given the implications of successful insurance coverage policies, it is important to understand whether implementation has translated into benefits for abortion care recipients. To answer this question, we interviewed abortion patients and key stakeholders (providers, clinic leaders, and advocates) to explore the ways in which Illinois policies have and have not been successful in reducing out‐of‐pocket costs for Illinois residents seeking early abortion care.

## METHODS

### Recruitment

Patients were eligible for the study if they spoke English, lived in Illinois, were between 18 and 45 years old, received abortion care in Illinois between March 2021 and February 2022, and had an abortion at or prior to 11 weeks of gestation (the gestational age limit for medication abortion in health centers where we recruited). We recruited participants based on order of outreach, with some purposive sampling at the end in order to reach thematic saturation around private insurance coverage. We sent flyers to 18 health centers providing abortion across Illinois, including centers that primarily provide abortion care and affiliated multiservice health centers providing full spectrum reproductive health services. Staff at health centers put up flyers and positioned smaller tear‐sheets in accessible locations in clinics, such as desk counters—they did not participate in further research activities. All posters were in English. Interested individuals were directed to complete an online screener and eligible respondents were contacted by the research team to schedule an interview. Patient recruitment ceased when the sample size reflected a roughly equal number of medical and instrumentation abortions and saturation around insurance coverage themes. Additionally, in 2021 we recruited key informants who were stakeholders in policy implementation to provide context for patient experiences, including state leaders in abortion advocacy, those involved in administrative matters or patient care, through network sampling and by word of mouth. Patient and key informant interviews were conducted between July 2021 and February 2022.

### Data collection

Research team members (LH, AM) conducted patient interview, offering patients the choice between video or phone. All patients preferred phone interviews. LH and AM identify as white, cis‐gender, heterosexual women earning middle‐high incomes. Verbal consent was obtained before conducting the interview and all participants received a $50 gift card after completing the interview. Patient interviews were audio‐recorded then transcribed by a third‐party transcription service. We created streamlined semi‐structured interview guides with input from leaders of the Chicago Abortion Fund to explore experiences using insurance and paying for abortion care, patient education and preferences around abortion method, obstacles and supports in obtaining abortion care, and impacts of the COVID‐19 pandemic on their experiences. Themes on method preferences and impacts of the pandemic are explored elsewhere.^25^ Interviews ranged from 15 to 30 min—the short length of the interview was due to the targeted nature of the data collection and a desire to reduce burdens for participation. Research staff verified all transcripts, removed any identifiable data, and uploaded them to Dedoose. Key informant stakeholder interviews were semi‐structured and tailored to the informant area of expertise but generally probed their perceptions of and experiences with implementation of new insurance coverage policies (Medicaid insurance coverage began in 2018 and private insurance coverage requirements started in June 2019). Stakeholder interviews were conducted by phone or zoom; interviews were audio‐recorded, transcribed, and de‐identified. The University of Chicago's Institutional Review Board approved all aspects of the study protocol.

### Analysis

Researchers created the codebook for patient interviews thematically, informed by the interview guide, previous literature, and insights from early interviews. The research team added agreed‐upon codes to the codebook. Initially, all members of the analysis team (LH, MQ, MB, and TT) coded the same three transcripts, then modified the codebook based upon incongruencies and emergent themes. Once the team established a sufficient level of coding concordance, three coders (MQ, MB, and TT) coded the remaining transcripts individually using Dedoose software. MQ, MB, and TT identify as cis‐gender, heterosexual women with low to middle incomes; two are White and one is Black. The researchers then created code summaries based upon emergent themes and trends in each code to facilitate in‐depth analysis and synthesis. With transcripts from key informant interviews, we created memos for each interview, guided by codes from the patient interview codebook, with representative quotations. We present themes related to patient experiences using insurance to pay for early abortion care.

## RESULTS

### Sample characteristics

We interviewed 50 patient participants; 25 identified as Black (50%), 16 as White (32%), nine as Hispanic (18%), and two as Biracial (4%) (Table 1). All participants identified as cis‐gender women. The majority of the participants were between the ages of 26–35. Almost all participants had some insurance at the time of procedure (Illinois Medicaid = 20, private = 28, out‐of‐state Medicaid = 1, no insurance = 1). Of the 20 participants with Illinois Medicaid, three were enrolled by meeting Medicaid presumptive eligibility (MPE) requirements at the time of their abortion. We also interviewed nine key informants as stakeholders with experience providing or billing for abortion or supporting insurance policy implementation in Illinois. Below we present salient themes that emerged from analysis; we have created tables that include description of the policies, findings from our study on the implementation of these policies, and recommendations based on our findings (Tables 4 and 5).

**TABLE 1:** Sample characteristics.

*n* = 50	*n* (%)
Age	
18–25	16 (32)
26–35	22 (44)
36–45	12 (24)
Mean (SD)	30.24 (6.56)
Type of procedure	
Medication	25 (50)
In‐clinic procedure	25 (50)
Insurance type	
Medicaid	21 (42)
Private	23 (46)
Dual private/Medicaid	4 (8)
No insurance	2 (4)
Race/ethnicity	
Black	25 (50)
White	16 (32)
Hispanic	9 (18)
Biracial	2 (4)
Geography	
Chicago	24 (48)
Cook County suburbs	10 (20)
Outside Cook County	16 (32)
Sexuality	
Heterosexual	47 (94)
Bisexual	2 (4)
Lesbian	1 (2)

### Higher rate of Medicaid coverage than private insurance

**FIGURE 1:**
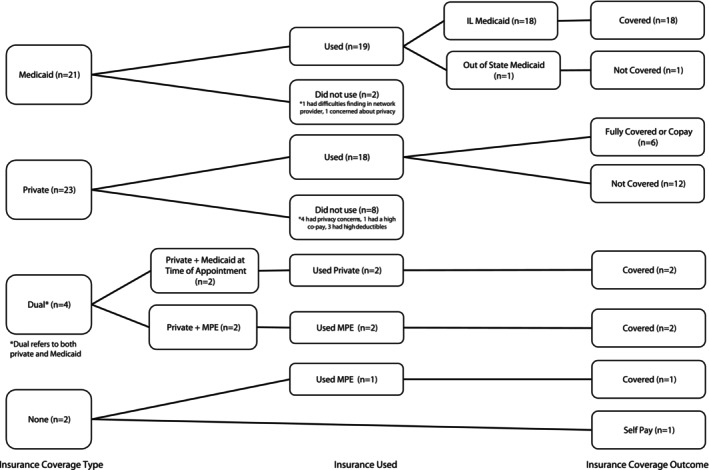
Insurance coverage by insurance outcome and number of participants.

Most participants who were insured by Illinois Medicaid received full coverage for their abortions (Figure 1). One‐third (*n* = 9) of Medicaid‐insured participants reported learning that their Medicaid insurance would cover their abortion at the time they made their appointment. The vast majority of participants (*n* = 20) with Illinois Medicaid had their abortions completely covered without cost‐sharing (Tables 2 and 3). Two patients with dual coverage (Medicaid and private insurance) used their private plans to cover their abortion. Two additional Medicaid‐insured patients elected to forgo insurance and paid out‐of‐pocket. The sole participant insured by an out‐of‐state Medicaid plan did not have her abortion covered by insurance.

**TABLE 2:** Insurance used to cover abortion.

Insurance type	*n* (%)
All (*n* = 50)	
Covered	30 (60)
Not covered	13 (36)
Not used	6 (14)
No insurance	2 (4)
Private (*n* = 28)	
Covered	9 (32)
Not covered	12 (43)
Not used	7 (25)
Medicaid (*n* = 23)	
Covered	20 (87)
Not covered	1 (4)
Not used	2 (9)

**TABLE 3:** Payment method for abortion by participant.

Payment type	*n* (%)
Paid at least some out‐of‐pocket (*n* = 23)	23 (47)
Chose to not use private insurance	5 (22)
Private insurance coverage was denied	11 (48)
Billed private insurance and paid some or all of appointment cost	4 (17)
Out of state Medicaid	1 (4)
Choose to not bill Medicaid insurance	2 (8)
Insurance completely covered abortion (*n* = 24)	24 (49)
Private	4 (17)
Medicaid	17 (71)
MPE	3 (12)

The most common outcome among participants with private insurance was that abortion was not covered by their insurance policy (Tables 2 and 3, Figure 1). Of the 26 participants covered by a private insurance policy, only one‐third (*n* = 8) had their abortions covered by their plans. Half (*n* = 13) said their abortion was not covered by their insurance plan and five chose not to use their insurance.

### Medicaid coverage communicated more clearly than private insurance

#### Medicaid coverage information and enrollment

In general, most Medicaid‐insured participants learned quickly that their plan covered abortion when they sought care. Under MPE provisions, health centers can screen and enroll eligible patients for Medicaid coverage that is available to pregnant patients in Illinois; if the patient is eligible, the patient is “presumptively” enrolled in Medicaid with coverage starting that day. Patients need to follow‐up and complete a formal Medicaid application. Overall, participants found the process of using Medicaid coverage fairly straightforward and accessible. According to one 33‐year‐old woman who had her instrumentation abortion covered, “everything was covered and they really took care of me at that facility”; she found the process satisfactory enough to recommend to others. Participants often reported learning that their Medicaid insurance would cover their abortion at the time they made their appointment, either by viewing information on the health center's website or when they contacted the health center; some participants learned of or gained coverage with MPE at the time of their appointment. One 24‐year‐old participant pointed out that information provided by Medicaid insurance plans should explicitly include details about abortion coverage so more people are aware, observing that current information “tell[s] you that if you're pregnant, you can get a free car seat, a phone if you can't call your doctor, but they don't tell you that, ‘Hey, if you need an abortion, we'll cover it at a bunch of places.’”

Five participants were offered MPE by clinic staff, either over the phone or at the time of service, two declined and three enrolled. Overall, participants found the MPE process to be smooth and thorough. A 33‐year‐old woman who was told about MPE over the phone, described her experience:

[the clinic] asked what I made annually and I told her and then she said that I would qualify for the Medicaid. So then when I went into the appointment and I talked to the individual ladies, they asked again about what I made monthly…and then the rest is… I applied.

Other participants echoed the ease of signing up for MPE both at the clinic and over the phone. However, two participants declined MPE citing concerns with delay in care, even though this fear while valid was likely unfounded. One participant seeking a medication abortion, who qualified but declined MPE found that being offered MPE at the time of appointment was challenging; she explained that “having to make that [insurance] decision on the spot was definitely stressful and harder.” The other participant who declined MPE echoed the fear of MPE causing medical delay: “I was afraid by the time that I got the insurance, and everything processed…that it would be too late [for medication abortion]. …so I just decided to go out‐of‐pocket.” Ultimately, the stress of thinking through insurance enrollment at the time of the appointment and concerns of no longer being eligible for preferred method choice, contributed to both of their decisions to pay out of pocket.

#### Information on private insurance coverage

Participants with private insurance faced a range of experiences when it came to finding out whether their abortion would be covered by insurance. Most relied on the health center to communicate with their insurance company to determine coverage. Many learned about their coverage status on the day of the appointment, which created a challenge for those who faced unexpected costs. Others had to navigate the need for referrals resulting from their HMOs with varying levels of ease.

Most participants reported that the health center directly contacted their insurance company on their behalf, a few also added that they reached out to the insurance company directly or read their policy online. Most participants who used private insurance were able to find out whether their abortion would be covered quickly, however, some faced challenges. Issues included miscommunication on referral needs and lack of coordinated communication between the patient, insurer, and clinic. For instance, a 38‐year‐old participant who opted for an instrumentation abortion noted that:

They [health center staff] just asked if I wanted to use insurance, and I told them I'd like to, but I didn't believe my insurance was going to cover it. They took my insurance information anyway, and just did due diligence on their end. So the day I showed up to my appointment, and I went through like the whole check‐in process, and [they] explain to me that my insurance provider was not going to cover it, and this is my total, and how did I plan to pay for it today?

A couple participants conveyed frustration at how difficult it was to find out information about their policy and a desire for increased transparency in what was covered by their plan. One such participant was a 43‐year‐old woman who expressed that:

It was really hard actually to find information. They [health center staff] tried to help. There was a little bit of feedback there. They tried to help but it was just like, it's so incomprehensible trying to go to the website and figure out like what's covered and why, and why not and things like that.

Participants also appeared to be affected by stigma, for example, another participant spoke about feeling “really uncomfortable” having “weird conversations with people that don't know me” at the insurance company in order to find out about private insurance coverage. Several spoke of having to secure a referral letter; one participant opted to pay cash rather than delay care and risk judgment or refusal, “…there's like concern over would the provider give me a letter to have that, you know?” While many participants indicated that health centers attempted to help to the best of their abilities with insurance navigation, more clarity on private insurance coverage was needed. A 24‐year‐old woman who received an instrumentation abortion reflected that “I wish [insurance information] was more known…[that] something like that is covered and that we have it at our disposal.”

### Privacy concerns prevented some from using insurance

Several participants cited specific concerns around privacy and confidentiality as factors in choosing to forgo their insurance coverage for abortion, most often with regard to private insurance plans. Privacy concerns were cited frequently when participants were on a parent's insurance plan, were concerned about employers' access to insurance statements, or were uncomfortable with paper documentation of their procedure. Several participants who were on their parent's insurance plan articulated concern around parents or guardians finding out about the abortion. A 25‐year‐old woman with private insurance reflected that, “I'm on my dad's insurance, but I did not want my dad or anyone to know”; this person planned to pay cash but was able to enroll in Medicaid through MPE.

Many participants were also concerned about “paper trails” and employers or others finding out about their abortion. A 36‐year‐old woman with private insurance reflected:

I guess, yeah. I don't really know how, I'm not very well‐versed in like what gets reported to the employer. I would imagine not a lot, but I'm not sure. And I guess just having less of a paper trail, so to speak, made me feel better. Even though I know that they couldn't do anything to me for doing it, but I just, again, I just started this job and I don't know. I just didn't want…I mean It's such a touchy subject that I just kind of didn't want them knowing about it.

Another participant with private insurance had a plan where their employer pays the first half of the deductible and “just didn't want to” involve insurance given the role of her employer.

Only one participant who had Medicaid insurance prior to their appointment cited privacy concerns as the main reason to pay the cost of their abortion out of pocket. This 21‐year‐old woman explained that “basically I didn't even mention that I had insurance, because I was afraid my parents would find out.”

### Successful abortion coverage reduced financial burdens and enhanced autonomy

Many participants who benefitted from insurance coverage for their abortion care expressed relief at having costs covered, gave examples of the financial burdens they were otherwise facing, and described feeling like they had more control over their abortion care choices. These themes were particularly prevalent among participants with Medicaid insurance coverage, which more uniformly covered all costs for a population with lower incomes.

Several participants described the importance of having costs covered. A 36‐year‐old woman with Medicaid explained the emotional relief she felt when learning that her abortion was covered: “[The cost] was one of my biggest concerns about getting an abortion done, because I would've had to pay all this money and so forth, but the Medicaid paid for it and so that was a big relief.” Another participant cited relief that all related costs were covered as well—including related prescriptions and pain medication. Many participants explained concerns around affording their abortion and the long‐term impact on their and their families' financial wellbeing prior to learning that their insurance covered their abortion. A 30‐year‐old woman and mother of four explained that before finding out she could enroll in Medicaid she “didn't have the extra $500 to get an abortion” and that she would “scrape it together somehow, but I would have been putting myself in a worse position.”

Other participants described concerns balancing bills, rent, and school costs if they needed to pay for their abortion out of pocket. Another participant who had private insurance that did not cover abortion care considered using a telehealth platform but observed that it “was pretty expensive online” to pay out of pocket; she was able to get MPE coverage at a clinic instead. A different participant with private insurance who did not use it due to a very high deductible was able to enroll in MPE; she talked about how coverage made her feel “good, given the situation, but definitely better knowing that I wouldn't have to come out of pocket with that amount because I really had no clue how I was going to do that.” A 27‐year‐old participant with Medicaid insurance further echoed the importance of affordable abortion access, highlighting all the other financial costs patients are juggling and the importance of decisional autonomy:

My insurance experience was amazing. I mean, just looking it up, they cover the important things … If I wasn't able to just be able to use my insurance, I would've had such a rough time financially to deal with all of this. Transportation and missing work and all of that. So having insurance made me feel like a lot more comforted … Because I didn't have to like be like, “Oh, I need money for an abortion.” Or I didn't have to tell anybody already about it. It was easy. I could just do it by myself.

Many participants felt that insurance coverage—public or private—allowed them to receive the care when they wanted, choose the method of care that they preferred, and felt more in control over the process. As one participant with private insurance coverage for abortion described, “It determined my scheduling. It also determined, because time is of the essence when this happens, it also made it easier. It took the stress off and the anxiety down for me having to prolong it and wait until I had the funds available.” A 24‐year‐old woman with multiple abortion experiences described the importance of early abortion access to reduce the burdens of pregnancy symptoms and stress: “I think part of why people, I think underestimate the value of early abortion care is because again, they don't understand both the physical and the mental toll that pregnancy takes on women.”

#### Challenges when abortion was not covered or coverage was limited

Conversely, those participants who did not experience straightforward coverage for abortion care—most of whom were privately insured—expressed feelings of stress, uncertainty, and constraint when reflecting on their insurance coverage. Lack of insurance coverage created financial burdens, affected the agency of many participants, and reduced participants' abilities to make choices around their abortion care. Many participants without coverage reflected that cost in some way influenced their abortion method choice, clinic choice, and timing of abortion. For example, a 24‐year‐old participant who received assistance from abortion funds described the stress of covering the cost,

That was the only thing that kept stressing me out because I also had rent due soon, and I was like, “What am I going to do? My rent is like $800 and this is $500. What am I going to do?” That was the primary thing that was stressing me out. And then once they said, “Oh, you're a [state university] student. It should cover it.” I was like, “Oh, good. I'm okay.” Then once I got to the clinic, it was like, “Oh, no, they actually don't.” I was like, “Oh, my God.” And I was like, “I can't pay for that. I can't.” So I was really concerned. I had made a choice that I was going to have it, the abortion, and then when they told me that it didn't cover it, I was literally, like, “I financially can't pay that right now.” But ultimately it did work out. But, yeah, that was the main thing that was stressing me out.

Some participants, such as a 21‐year‐old woman, felt that “in retrospect, if I could have afforded it or if my insurance would've covered it, I would've definitely chosen the surgical one.” Medication abortion is often cheaper than an instrumentation abortion, therefore, some participants had to choose medication abortion methods due to financial reasons. Several participants noted delays in receiving abortion care due to anxieties around insurance coverage and issues with finding health centers that accepted their insurance. Another participant with student health insurance through a state university that would not cover abortion opted for a medication abortion and explained how the cost of the abortion resulted in delay despite her decisional certainty, reflecting:

When I found out that I was pregnant, I knew, I was like, ‘I can't.’[…] But when I saw how much it was, I was like, ‘There's no way. I can't even afford that right now.’ So I literally waited three or four more weeks.

Furthermore, several participants described private insurance policies around referrals, reasons for abortion, type of procedure, and high deductibles as limiting any benefits of insurance coverage. For example, a 30‐year‐old woman with private insurance explained that she had intended to use her insurance to cover the cost of the appointment but, “when I went in for the appointment she [the staff person] informed me that because I hadn't met my deductible it wasn't going to cover, I think elective procedures, I think she said. So I would just be paying out of pocket.” A 23‐year‐old woman with private insurance explained that she had to pay out of pocket because she was told “they [the insurance company] didn't cover surgery and it had to be early stages of abortion, six weeks, I believe”; she also further included that “there were stipulations too if they could cover the pill. It was a very conservative point of view. So if it was rape or a medical emergency or something.”

### Stakeholder perceptions of insurance policy implementation

#### Medicaid coverage

Speaking from an implementation perspective, the majority of key informants found current Medicaid abortion coverage to be successful in reducing financial burdens some people face when obtaining abortion care. A person who works in billing at multi‐service health center felt that MPE in particular was “going really, really good.”

While Medicaid coverage appears to be effective for patients, providers described issues related to Illinois' use of managed care plans to facilitate Medicaid coverage; providers and operations leaders reported challenges with communication about benefits and clarity on coverage. For instance, some key informants described that Medicaid‐insured patients often do not know that they have coverage for abortion or that they've received incorrect information from their plans. As one informant described,

One of the times a patient did call me and I ended up calling. She ended up getting the reference number. Which is very smart of her. And I ended up calling one of the [Medicaid managed care] payers. And the payer stated, ‘Yeah, we don't cover it.’ I was like, ‘But Medicaid does. And that's something that you should [tell] the patient.’ And they're just like, ‘Oh, it's not.’ They're not really apologetic. And it's basically like, ‘Well, we don't. So that's all that we're going to relay.’

Separating the medical expenses that needed to be paid by Medicaid managed care (Hyde allowable abortions) and by Medicaid fee‐for service (non‐Hyde allowable) also created work for clinics; as one key informant at an abortion clinic noted, “I think it's just, it's harder on the billing side than it needs to be. If … it is one of the Medicaid [plans] that we kind of contract with, that has to be sent either to the HMO versus the central Medicaid if it's a medically indicated reason.”

Further, while informants welcomed Medicaid reimbursement rate increases, they also outlined some ongoing challenges, including additional paperwork, complications for patients with both Medicaid and Medicare, and concern regarding delays in Medicaid reimbursement. As one informant observed, “we are relying on Medicaid to pay us in a timely fashion”, which is very different from when patients used to “just put the money on the desk.”

Finally, one abortion provider felt that while Medicaid coverage of abortion was essential, “the procedure was still very costly to a lot of people,” explaining that:

I think the barriers… I would say they all fall under the umbrella of poverty. I mean, the truth is that people seeking abortion are now able to undergo the procedure at no cost … That doesn't mean… that the experience is cost‐free, right?

They go on to note that patients may face costs related to transportation, missed work, and childcare needs. Nonetheless, this provider felt that the Medicaid insurance expansion was important when reflecting on a recent patient, “she was very, very early in the pregnancy but it was very clear… it [the abortion] being free was extremely important to her. She probably wouldn't have been there that day if she had had to look for money to pay for it.”

#### Private insurance coverage (information on private insurance coverage)

Echoing patient reporting, many key informants expressed frustration and confusion around the implementation of policies requiring private insurance coverage of abortion, pointing to difficulties determining which plans must comply and specific terms of coverage. In part, this arises because the state law only governs some insurance plans; federal law governs others and it is not always clear to patients and providers which law applies. The director of a regional advocacy organization explained that at the initial rollout of the requirement:

We were hearing from a lot of people, both individuals and from health centers, that private insurance was not covering abortion care the way it should. As we've gone on, and we have tried to do some advocacy in our office too, that is very difficult for us to advocate…when somebody calls us and says, my private insurance isn't covering, or if a clinic calls us we don't have authorization to talk on behalf of a patient. So it's hard to just determine what's going on.

While this key informant explains that some of these issues have been resolved with time, concerns and confusion around private insurance coverage of abortion continue. For instance, abortion providers report that some private insurance plans may only cover abortion in certain circumstances, such as limiting the number of abortions covered or only offering coverage for undefined “therapeutic” reasons. One provider explained the consequences of confusion around private insurance coverage, “they [the insurance company] had given our pre‐authorization person the approval and then after [the abortion was completed] said, ‘Oh, just kidding. It's not covered.’” This patient was charged $20,000 and had to advocate for over a month to get the charge removed. While relieved that the situation was resolved, the provider felt that “they [the insurance company] just don't know about the law yet.” Another provider pointed out that Illinois law does not create a clear mechanism for enforcement, noting the law “doesn't really specify on how we can fight with the insurance company to have this get paid for the patient. There's no … instructions or policy.”

## DISCUSSION

Our study findings highlight the efficacy of Medicaid coverage and demonstrate potential gaps in implementation of the private insurance requirements. While other studies have explored the efficacy, accessibility, and acceptability of insurance coverage for abortion for both patient and provider, few studies have qualitatively explored patient perspectives to capture nuances regarding use of insurance to cover abortion in a state requiring both Medicaid and private insurance coverage.^9^, ^26^, ^27^ Other studies have demonstrated that those without Medicaid coverage have a longer time between deciding to have an abortion and receiving an abortion than those who do have Medicaid coverage of abortion.^28^ Further, research has found that private insurance denials of reproductive healthcare occur at a higher rate for those with lower income.^29^

As reflected in other studies, our study expands and illustrates how barriers associated with finances and insurance decrease the reproductive autonomy and agency of people seeking reproductive healthcare.^7^, ^30^, ^31^ Lower levels of reproductive autonomy can negatively affect an individual across their life‐course, by continuing cycles of poverty and increasing socio‐demographic inequities.^12^, ^32^, ^33^, ^34^ While participants in our study ultimately were able to access abortion, for many with private insurance it was not without significant burdens. This study also further supports how typical structural barriers of health care costs such as referrals, co‐pays, and high deductibles are compounded by abortion exceptionalism (e.g., insurance often does not cover abortion like other health care) and stigma, which increases the marginalization and inaccessibility of abortion.^12^, ^32^, ^33^, ^34^ The structural implications of inequitable insurance coverage are often compounded by other oppressive factors (racism, classism, religious extremism, Hyde Amendment, etc.).^22^, ^35^, ^36^, ^37^, ^38^, ^39^

Our findings suggest that successfully implemented state Medicaid coverage does reduce some structural financial barriers for those with lower incomes who can access Medicaid. Part of successful implementation includes ensuring providers can participate in the Medicaid program with straightforward processes and appropriate reimbursement. Further, state Medicaid coverage has helped to circumvent issues with individuals who have both Medicaid and Medicare (see Table 4). Medicaid expansion eliminates out‐of‐pocket payments for qualifying individuals when accessing abortion care, thus increasing reproductive autonomy for those with limited financial capital.^6^, ^10^ However, when reviewing virtual‐only medication abortion providers serving Illinois on abortionfinder.org, their websites suggest that most do not accept private insurance or Medicaid or participate in MPE.^41^ In particular, the ease with which patients have been able to enroll onsite at the time of appointment as health centers have successfully implemented presumptive eligibility screening has helped ensure the benefits of coverage extend to all who should have access. This finding contrasts with the more prevalent experiences of abortion scarcity and “unchoosabilty” experienced by those living on or below the federal poverty guidelines.^33^ Abortion unchoosabilty is defined as a person feeling unable to choose an abortion due to reasons such as structural obstacles (e.g., cost, marginalization of abortion), cultural stigma (e.g., morality), and personal healthcare experiences (e.g., medical trauma).^33^, ^38^, ^39^, ^40^ While barriers persist for Medicaid recipients in Illinois, expansion of Medicaid helps to mitigate one aspect of unchoosabilty for those living at or below the federal poverty guidelines. Our study found that for those who are Illinois Medicaid eligible, not only is abortion an option, but the factors around abortion (such as: location, timing, method) shift into the hands of the patients with the removal of the cost of the procedure.

**TABLE 4:** Status of Illinois Medicaid policy for abortion coverage.

Topic	IL Medicaid policy language	Problem identified	Status of implementation
Presumptive Eligibility Enrollment (MPE)	“Medicaid Presumptive Eligibility (MPE) offers immediate, temporary coverage for outpatient healthcare for pregnant women.^a^”	MPE allows for pregnant people to enroll and receive abortion care on the same day. This allows pregnant people to be enrolled briefly in Medicaid just for the coverage of their abortion.	MPE seems to be largely successful for both provider and patient.
Appropriate Reimbursement Rates	“Effective with dates of service beginning September 1, 2022, rates for surgical abortion and medication abortion services will be increased as follows:” Medication: $558 D&E: $1920 D&C: $792^b^	Prior to Medicaid expansion of abortion coverage, reimbursement rates had not been updated for decades, leaving abortion providers faced with a financial deficit when accepting Medicaid coverage of abortion. On September 1, 2022, IL increased Medicaid Reimbursement rates to better cover the cost of all types of abortions.	Illinois now provides some of the highest Medicaid reimbursement rates for abortion in the country.
Electronic Claims Filing	“The HFS 2390 Abortion Payment Application is being obsoleted and the Department will no longer require it for claims received on and after November 1, 2019… Therefore, claims containing abortion procedures must be billed electronically beginning November 1, 2019.^c^”	Prior to 2019 any abortion related Medicaid claims had to be filed by paper. This posed a barrier in terms of both access and timely reimbursement for some clinics. In 2019 providers can file their claims electronically.	This process has been rollout successfully.
Dual Medicaid/Medicare Enrollment	“August 1, 2022, providers will not be required to bill Medicare as the primary insurance for abortion procedures for MMAI plan customers (dual Medicare and Medicaid coverage)^b^”	Most abortion providers are not Medicare providers, therefore, patients with dual enrollment would sometimes not be able to use their Medicaid to cover the cost of abortion care. Providers now have the option to bill Medicaid first which will allow MMAI customers to use Medicaid to cover the cost of their abortion.	This is a newer development and we do not yet have enough data to assess the successfulness of this implementation.
MCO Communication	“Managed Care Organizations (MCOs) that help administer Medicaid that they are contractually required to include information on abortion service coverage in their member handbooks.^d^” A notice was issued to Managed Care Organizations (MCOs) that help administer Medicaid “that they are contractually required to include information on abortion service coverage in their member handbooks” and ensure call center staff are prepared to answer questions on abortion coverage.	Specific language and training were included for MCO employees to assist with the change in coverage policies. Enrollees were not given information on abortion coverage and benefit terms; MCOs were instructed to include this information in handbooks and train call center staff and on abortion coverage policies.	Our research indicates that there are still issues with MCO communication despite having language in place to help facilitate MCO usage for abortion care. Our research indicates that there were issues with MCO communication prior to this notice; more research is needed to determine if this action has improved communication and transparency.

^a^ HFS, “Moms and Babies”. Accessed on March 6, 2023. Available at: https://www2.illinois.gov/hfs/MedicalPrograms/AllKids/Pages/MomsAndBabies.aspx#MPE.

^b^ Medicaid bulletin, “Abortion Services – Rate Increase and Billing Change,” September 6, 2022. Accessed on March 6, 2023. Available at: https://www2.illinois.gov/hfs/MedicalProviders/notices/Pages/prn220906a.aspx.

^c^ Medicaid Bulletin, “Changes to Claims Submittal Process and Rates for Abortion Procedures,” November 1, 2019. Accessed on March 6, 2023. Available at: https://www2.illinois.gov/hfs/MedicalProviders/notices/Pages/prn191101b.aspx.

^d^ HFS Official Letter, “Pritzker Administration Affirms State Coverage of Abortions in Comprehensive Healthcare for Pregnant Women,” 5/12/22. Accessed on March 6, 2023. Available at: https://www2.illinois.gov/IISNews/24885‐Pritzker_Administration_Affirms_State_Coverage_of_Abortions_in_Comprehensive_Healthcare_for_Pregnant_Women.pdf.

However, Medicaid access covers only the cost of the procedure – leaving other factors such as transportation or childcare costs as a significant barrier for some. Further, our study highlights the benefits of Medicaid coverage can be realized only after successful implementation.^6^, ^7^ In Illinois, health centers actively participate in the Medicaid program because they want to see patients get affordable care but also because reimbursement rates cover costs of care, processes are in place for timely reimbursement, and health centers can easily enroll patients through the MPE program.^6^ While participants may have been concerned about MPE at the time of roll out, key informants have indicated this process has improved.

At the same time, our study further demonstrates and confirms that many private insurance plans are failing to cover costs associated with abortion care.^24^, ^27^, ^42^ Participants cited confusion around covered care, concerns around privacy, high deductibles, lack of referrals, restrictions on methods, and unclear communication as primary reasons that they did not or could not use their private insurance coverage. These findings demonstrate that the pathway to private insurance coverage of abortion is difficult even within a state that has enacted proactive policy. In part, this stems from state insurance laws—the Illinois requirement to cover abortion only applies to plans governed by state law (rather than federal laws such as the Employee Retirement Income Security Act [ERISA]). It is very difficult for both patients and providers to determine if any given insurance plan is not complying with state law so there is little accountability when a plan fails to cover abortion but should be doing so. Mechanisms exist to address this issue; for instance, Maryland has implemented a requirement for state insurance plans to indicate they are bound by state law on an individual's insurance card.^43^ Integrating a policy like this may improve transparency and accountability for insurance plans.

Finally, with both Medicaid and private insurance coverage, some participants feared that using insurance would lead to a breach of privacy—either with parents or other family members or with employers. The fear around privacy and potential breach of confidentiality may be heightened due to abortion stigma and confusion around abortion legality.^44^, ^45^, ^46^

Our study findings are essential as successful implementation of Medicaid and private insurance coverage policy may translate to better resource allocation for those without coverage.^9^ This is particularly salient for states such as Illinois which must now support health centers in providing care for many individuals from out of state and who may be fearful of a privacy breach.^1^

Other states seeking to improve abortion access could model their programs on current Illinois Medicaid policy, paying particular attention to seamless presumptive eligibility enrollment and appropriate reimbursement rates. For participants with private insurance, however, experiences varied greatly with many still having to pay out of pocket for their care. Further investigation may be needed to understand the level of compliance among private insurance companies and to identify mechanisms for enforcing state law requirements (e.g., perhaps reviews by the Department of Insurance).

### Limitations

Strengths of our study include our diverse sample of participants who had experiences with both medication and instrumentation abortion and our large qualitative‐study sample. Limitations include the fact that we only spoke with participants who sought early abortion and not abortions later in gestation, which may incur greater costs (both with and without coverage). We also only spoke to those who were actually able to obtain a desired abortion; we do not know how insurance may have affected those who were unable to obtain an abortion. Our study was also conducted during the COVID‐19 pandemic and our recruitment methods were limited to handouts and flyers. This approach required participants to self‐select into the study and have access to a phone and secure location for the interview, which may have introduced bias within our sample. Finally, participants were not evenly distributed from the health centers. We recruited more than half from a large, affiliated health center and the rest from three different non‐affiliated abortion clinics. This skewed distribution may have affected the makeup of our sample.

## CONCLUSION

When abortion was fully covered by public or private insurance, it reduced financial burdens and enhanced reproductive autonomy by ensuring patient choice around timing, method, and location of the abortion. In this study, we observed that Medicaid coverage facilitated by HB40 is an effective mechanism for eligible participants to gain access to affordable care and exercise choice in their abortion experience (Table 4). Problems with transparency in private insurance could possibly be addressed by requiring private insurers to clearly include abortion coverage information in member handbooks and train staff on abortion‐related benefits, similar to requirements the state recently made of Medicaid managed care plans (Table 5). Lastly, repealing the Hyde Amendment would help to alleviate some of the structural barriers preventing equitable abortion access across the country.^47^, ^48^

**TABLE 5:** Status of Illinois private insurance coverage policy for abortion care.

Topic	State law summary	Problem identified	Recommendation for implementation
Abortion coverage equivalent to pregnancy care coverage	“…no individual or group policy of accident and health insurance that provides pregnancy‐related benefits may be issued…in this State…unless the policy provides a covered person with coverage for abortion care.” (215ILCS5/356z.4a)	Patients and key informants had difficulty confirming inform‐ation about abortion coverage and understanding whether plans were in or out of compliance.	State agencies should establish mechanisms to review and ensure compliance with coverage requirements. For example, insurance plans could be required to indicate they are governed by state law on an individual's insurance card, similar to Maryland
Appropriate cost‐sharing	“Coverage for abortion care. May not impose any deductible, coinsurance, waiting period, or other cost‐sharing limitation that is greater than that required for other pregnancy‐related benefits covered by the policy.” (215ILCS5/356z.4a)	Patients reported high deductibles and/or co‐pays even if insurance covered abortion care, keeping out‐of‐pocket costs high.	However, given the importance of pregnancy‐related care, the state should explore removing cost sharing (co‐pays and deductibles) for all pregnancy‐related care, including abortion, comparable to Illinois Medicaid.
Abortion coverage under all circumstances	“Except as otherwise authorized under this Section, a policy shall not impose any restrictions or delays on the coverage required under this Section.” (215ILCS5/356z.4a)	Patients and providers reported limitations on coverage based on reason for or circumstances of abortion	It is difficult to determine whether plans are governed by state law. State law or regulatory action could affirm that all abortion care should be covered equally.
Insurance plan communication	No policy language	Patients and providers reported difficulty getting clear information from insurers about covered benefits regarding abortion care.	State law should require private insurers to include abortion coverage benefits information in their member handbooks, similar to Medicaid requirements for managed care organizations in Illinois.
Confidential care	No policy language	Patients reported forgoing use of private insurance because they wished to keep their abortion care confidential from others on the insurance plan.	State law should require mechanism by which patients can request that Explanation of Benefits and other insurance information for sensitive services be sent to a preferred address or contact, similar to Illinois Medicaid policy.
